# Molecular Survival Strategies Against Kidney Filtration: Implications for Therapeutic Protein Engineering

**DOI:** 10.3390/biophysica6010004

**Published:** 2026-01-13

**Authors:** William P. Heaps, Anne Elise Packard, Kristina M. McCammon, Tyler P. Green, Joseph P. Talley, Bradley C. Bundy, Dennis Della Corte

**Affiliations:** 1 Department of Physics and Astronomy, Brigham Young University, Provo, UT 84602, USA; 2 Department of Chemical and Biological Engineering, Brigham Young University, Provo, UT 84602, USA

**Keywords:** glomerular filtration, protein therapeutics, half-life extension, PEGylation, Fc-fusion, pharmacokinetics, albumin binding, FcRn recycling

## Abstract

The glomerular filtration barrier poses a significant challenge for circulating proteins, with molecules below ~60–70 kDa facing rapid renal clearance. Endogenous proteins have evolved sophisticated evasion mechanisms including oligomerization, carrier binding, electrostatic repulsion, and FcRn-mediated recycling. Understanding these natural strategies provides blueprints for engineering therapeutic proteins with improved pharmacokinetics. This review examines how endogenous proteins resist filtration, evaluates their application in protein engineering, and discusses clinical translation including established technologies (PEGylation, Fc-fusion) and emerging strategies (albumin-binding domains, glycoengineering). We address critical challenges of balancing half-life extension with tissue penetration, biological activity, and immunogenicity—essential considerations for the rational design of next-generation therapeutics with optimized dosing and enhanced efficacy.

## 1. Introduction

The therapeutic potential of protein and peptide drugs is often constrained by their short systemic half-life. Rapid renal clearance of small proteins (<60–70 kDa) necessitates frequent dosing, driving up treatment costs and reducing patient adherence [1,2]. This filtration threshold imposes a major barrier for many modern biologics, such as cytokines, enzymes, antibody fragments, and nanobodies, which fall below the glomerular cutoff [3,4]. While other mechanisms of clearance also dictate a protein’s systemic half-life, this review focuses primarily on the molecular strategies that combat rapid renal filtration. As the field of biologics expands toward smaller, more specialized formats, rational strategies to control pharmacokinetics have become increasingly important.

**Figure 1.**
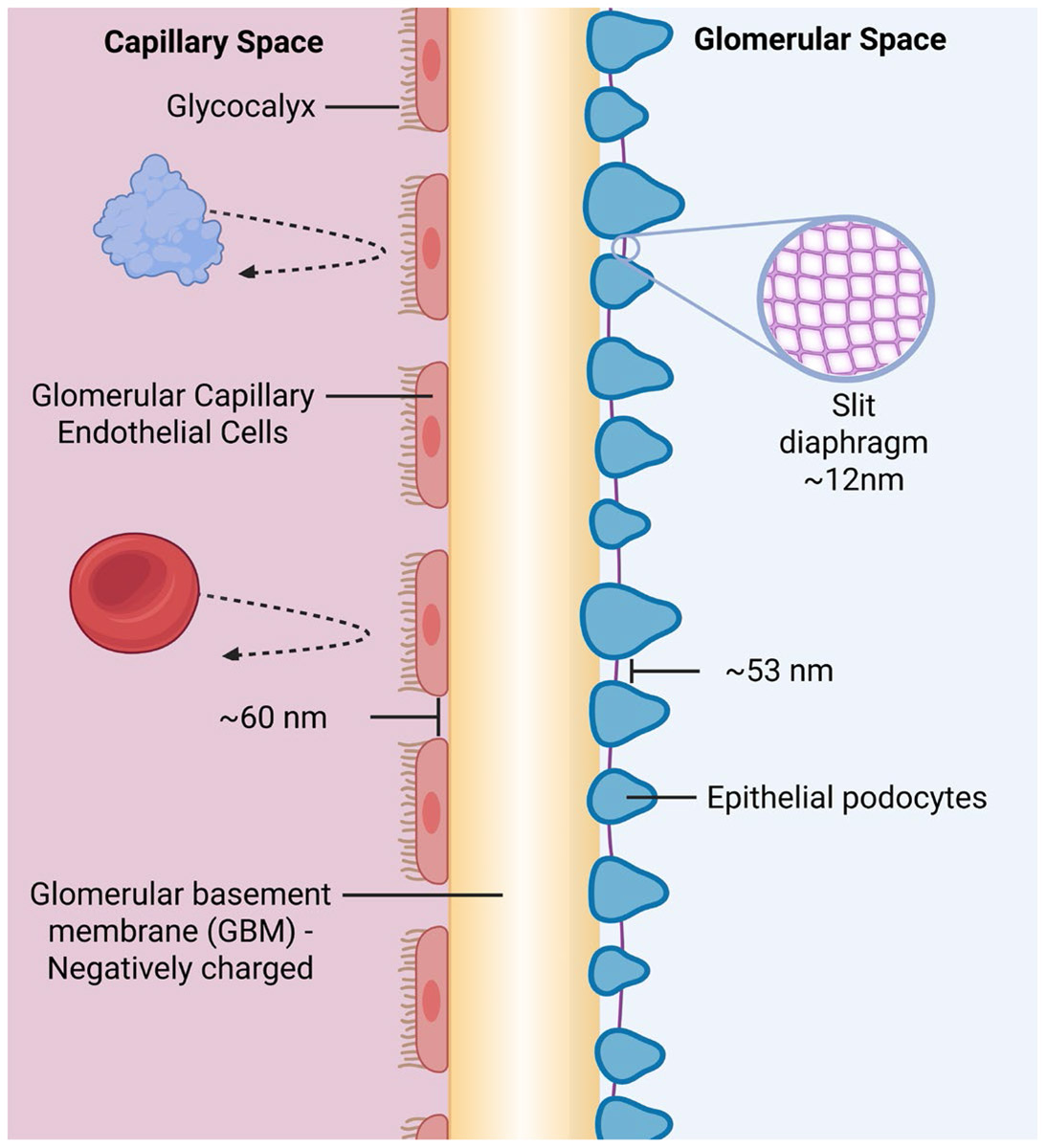
Schematic representation of the glomerular filtration barrier. The barrier comprises three major structures: the fenestrated endothelial layer coated with a negatively charged glycocalyx, the trilaminar glomerular basement membrane, and the podocytes interconnected by the slit diaphragm. Although the schematic displays the approximate geometric dimensions of the fenestrae, podocyte, and slit diaphragm spacing, these gaps do not reflect the true functional pore size. Instead, effective filtration permeability is determined by the combined membrane architecture, charge distribution, and fluid-dynamic properties of all three layers, resulting in nanometer-scale functional pores that permit water and small solutes through while restricting albumin and larger plasma proteins [5].

The glomerular filtration barrier (GFB) is the primary determinant of renal protein clearance (see Figure 1). Together, the layers of the GFB exhibit both size and charge selectivity: molecules larger than ~70 kDa or carrying strong negative charge are typically retained in circulation [6,7]. The podocyte slit diaphragm serves as the principal size-selective barrier and it also possesses electrostatic properties that repel negatively charged molecules [8]. In contrast, the glomerular basement membrane functions as a secondary filter that restricts the passage of larger macromolecules and exhibits minimal charge selectivity [9]. The fenestrated endothelium provides additional filtration by size and charge selectivity [10]. Consequently, small or cationic therapeutic proteins are rapidly filtered, leading to short half-lives and limited bioavailability [11].

Therapeutics that are too large to be filtered renally are cleared via other pathways. For instance, those with exposed regions or conformational flexibility may be subject to proteolytic degradation by circulating or tissue-associated proteases. Other molecules are internalized via receptor-mediated endocytosis and are directed to lysosomes for degradation. Molecules that bear hydrophobic patches or non-native glycan structures are frequently cleared through hepatic uptake. In addition, therapeutics that elicit immunogenic responses are rapidly cleared by anti-drug antibodies that form a complex with the therapeutic, promoting Fc receptor-mediated uptake and phagocytosis. While these pathways are important to consider in designing biologics, renal filtration is the primary concern of many modern protein and peptide therapeutics due to their small size.

Nature provides blueprints for overcoming renal filtration. Endogenous proteins employ size expansion, carrier binding, and recycling mechanisms to prolong circulation. Examples include reversible homo-oligomerization (as in transthyretin), binding to larger carrier complexes (insulin-like growth factor (IGF)–IGF binding protein (IGFBP)–acid-labile subunit (ALS)), and FcRn-mediated recycling that protects immunoglobin G (IgG) and albumin from degradation [12-16]. Charge-based mechanisms further enhance persistence. For example, the dense negative charge and terminal sialylation of many plasma glycoproteins minimize filtration and receptor-mediated clearance [17]. These strategies demonstrate evolutionarily optimized solutions to pharmacokinetic challenges that now inspire therapeutic engineering.

Translating these natural principles has led to numerous half-life extension technologies. Established approaches, such as PEGylation and Fc-fusion, enhance molecular size or exploit FcRn recycling, while emerging methods leverage albumin-binding domains, polysialylation, PASylation, and glycoengineering to achieve similar effects with greater control [18-23]. However, prolonging circulation introduces trade-offs: increased size can impair tissue penetration, and chemical modifications may reduce biological activity or raise immunogenicity [24,25].

This review explores how insights from endogenous protein behavior inform the design of long-circulating therapeutics. Here, we examine natural survival mechanisms including size-based and charge-based strategies, while also highlighting engineered approaches such as PEGylation, Fc-fusion, and albumin- or glycan-based methods. Additionally, we discuss key design challenges and consider emerging directions toward next-generation, long-acting biologics. While previous reviews have described strategies for extending the half-life of biologics [26,27], this review uniquely grounds these approaches in the anatomy and physiology of the renal filtration barrier to explain why they are effective. It further compares the strategies with endogenous survival mechanisms and highlights how emerging computational tools may be leveraged to further extend the circulatory half-life of therapeutics in vivo.

## 2. Natural Survival Mechanisms

**Figure 2.**
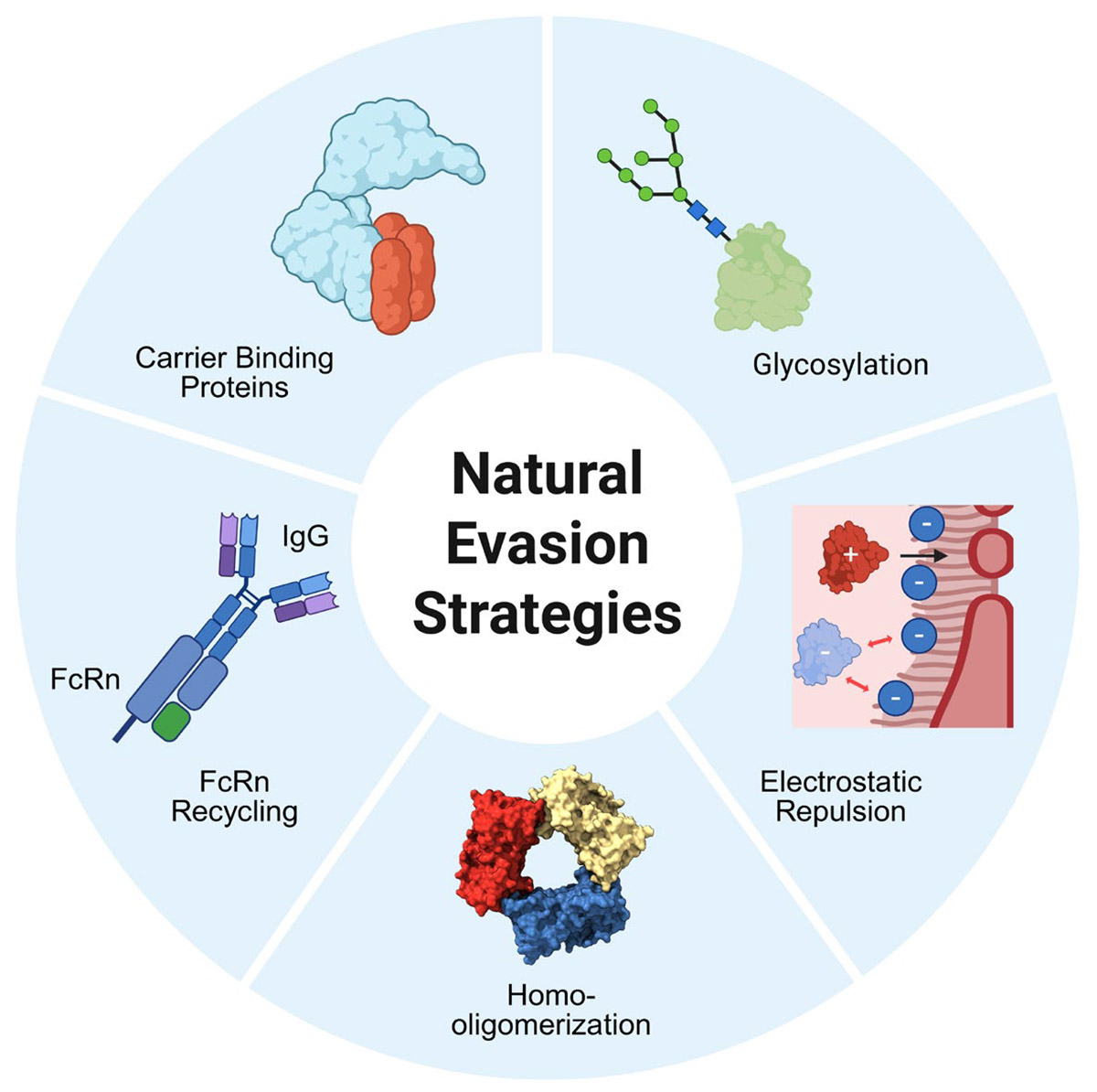
Overview of natural protein survival strategies that limit renal filtration. Illustrated are five endogenous mechanisms that extend the circulatory half-life of plasma proteins. Size-based strategies include homo-oligomerization, which increases the hydrodynamic radius, and reversible binding to high-molecular-weight carrier proteins. FcRn-mediated recycling salvages IgG and albumin in endosomes, returning them to circulation independent of glomerular size limits. Charge-based strategies include electrostatic repulsion from the negatively charged filtration barrier and glycosylation, particularly sialylation, which increases the hydrodynamic volume and net negative charge [28].

Endogenous plasma proteins face the same environment as therapeutic biologics, yet many have evolved sophisticated adaptations that enable prolonged circulation. These mechanisms can be broadly classified into size- or charge-based strategies that modulate the protein’s interaction with the glomerular filtration barrier, or receptor-based strategies that protect the protein from lysosomal degradation (see Figure 2). Understanding these natural adaptations provides a physiological foundation for efforts to engineer proteins with enhanced pharmacokinetic stability.

### 2.1. Size Expansion Strategies

#### 2.1.1. Homo-Oligomerization

Homo-oligomerization, the self-association of identical protein subunits, substantially influences a protein’s pharmacokinetics by expanding its hydrodynamic size and thereby reducing renal clearance [29,30]. Defined interfacial motifs—including β-sheet extension, coiled-coil elements, and metal-ion coordination—stabilize higher-order assemblies that exceed the glomerular filtration threshold. This structural amplification limits sieving through the renal barrier and enhances plasma retention in a manner analogous to macromolecular conjugation strategies. In addition to added size and mass, oligomeric interfaces can stabilize protein conformations against proteolysis or receptor-mediated endocytosis, further prolonging circulatory half-life [31]. As a result, homo-oligomerization represents a natural mechanism by which proteins maximize both biophysical stability and systemic persistence.

Adiponectin provides a physiological model of this principle. Circulating adiponectin forms trimers, hexamers, and high-molecular-weight multimers through inter-subunit disulfide bonds, particularly involving Cys39. In vivo tracking of fluorescently labeled recombinant adiponectin demonstrated rapid clearance of trimeric species compared to high-molecular-weight assemblies, with the Cys39 mutant displaying near-immediate loss from circulation [32].

#### 2.1.2. Carrier Protein Binding

Many small endogenous proteins and peptides evade rapid glomerular filtration by forming high-molecular-weight complexes with dedicated carrier proteins, a strategy that dramatically expands hydrodynamic volume while preserving a reservoir of free ligand for biological activity [33-35]. This reversible association transforms molecules that would otherwise exhibit half-lives of minutes into circulating species that persist for hours to days, effectively mimicking the pharmacokinetic protection conferred by covalent size expansion.

The insulin-like growth factor (IGF) system exemplifies this principle. Free IGF-I and IGF-II are highly susceptible to renal clearance, leading to circulating half-lives of less than 10 min [36]. In plasma, however, more than 99% of IGFs exist in binary or ternary complexes with insulin-like growth factor-binding proteins (IGFBPs) and, for IGFBP-3 and IGFBP-5, the acid-labile subunit. The resulting 150-kDa ternary complex (IGF + IGFBP-3/5 + ALS) far exceeds the glomerular size threshold, extending half-life to approximately 12–16 h [37-39]. Structural studies have revealed that ALS stabilizes the ternary assembly through extensive contacts that lock IGF in a conformation incompatible with receptor engagement, while also preventing undesired cross-reactivity with the insulin receptor—a critical safeguard given the high circulating concentrations of IGFs [39,40].

Thyroid hormones employ an analogous carrier-mediated strategy. More than 99.9% of thyroxine (T4) and triiodothyronine (T3) circulates bound to thyroxine-binding globulin, transthyretin, and albumin, extending the half-life of T4 to approximately seven days while maintaining only a minute free fraction available for tissue uptake and receptor signaling [41]. This tight binding ensures stable hormone delivery across physiological fluctuations, illustrating how carrier association not only restricts renal loss but also establishes a dynamic equilibrium that regulates bioactivity [42].

These natural carrier systems—IGF-IGFBP-ALS and thyroid hormone-binding proteins—are examples of systems that act as a reservoir for their respective ligands. In such systems, the binding affinities of the carrier proteins are higher than the receptors’. This thermodynamic profile creates a stable circulating pool with slow dissociation rates, buffering plasma concentrations against rapid fluctuations [41,43]. In contrast, other carrier systems are characterized by lower carrier protein affinity and higher receptor affinity, establishing a gradient that promotes rapid dissociation and bioavailability. These differences exhibit how nature utilizes variations of binding thermodynamics to accomplish different purposes and give insight into how these systems might be engineered according to the desired outcome. Despite these variations, both strategies share a major pharmacokinetic benefit: sequestering the ligand within a macromolecular complex that exceeds the glomerular filtration threshold.

### 2.2. Receptor-Based Mechanisms

#### FcRn-Mediated Recycling

The neonatal Fc receptor (FcRn) provides an effective endogenous mechanism for extending protein half-life that operates independently of size or charge selectivity at the glomerulus [29-31]. Expressed on endothelial cells, hematopoietic cells, and many epithelial barriers, FcRn binds both IgG and albumin in a strictly pH-dependent manner: high-affinity interaction occurs only at an acidic pH (<6.5) within the sorting endosome, driven by protonation of histidine residues on the ligand that engage acidic pockets on FcRn, whereas binding is negligible at neutral extracellular pH [44,45].

Following fluid-phase pinocytosis, IgG and albumin are sequestered into early endosomes. There, at low pH, they bind FcRn on the vesicular membrane; unbound proteins are trafficked to lysosomes for degradation, while FcRn-bound ligands are protected and returned to the cell surface in recycling endosomes. Upon vesicle fusion with the plasma membrane, exposure to neutral pH triggers immediate ligand release back into circulation [46,47]. This highly efficient salvage pathway recycles the majority of internalized IgG and albumin, conferring serum half-lives of approximately 21 days for both proteins—far longer than expected from size alone.

The physiological importance of FcRn is illustrated by familial hypercatabolic hypoproteinemia, in which FcRn mutations abolish recycling and reduce IgG and albumin half-lives to <5 days, leading to profound hypoalbuminemia and immunodeficiency. Conversely, certain small endogenous molecules exploit the system indirectly: long-chain fatty acids, bilirubin, and some hormones bind non-covalently to albumin and thereby “hitchhike” on its FcRn-mediated protection despite their own minuscule size [48,49].

This natural recycling mechanism has become a cornerstone of therapeutic protein engineering. By conferring pH-dependent FcRn binding—whether through Fc fusion, albumin fusion, albumin-binding domains, or direct mutation of the therapeutic itself—modern biologics can achieve half-lives approaching or exceeding those of native IgG, transforming dosing regimens from daily to weekly or even monthly (Section 3). As one of nature’s most effective methods for half-life extension, FcRn recycling has become a central focus of current half-life extension strategies.

### 2.3. Charge-Based Mechanisms

#### 2.3.1. Electrostatic Repulsion

The glomerular filtration barrier’s negative charge creates an electrostatic barrier to anionic macromolecules, representing a critical determinant of protein clearance beyond size selectivity alone [50-52]. This charge-based discrimination arises from negatively charged glycosaminoglycans, particularly heparan sulfate proteoglycans, distributed throughout the endothelial glycocalyx, glomerular basement membrane, and podocyte surfaces. Given equivalent size, cationic proteins filter more readily than neutral or anionic proteins, demonstrating that charge profoundly influences glomerular permeability [53].

The molecular basis of charge selectivity involves electrostatic repulsion between anionic proteins and fixed negative charges within the filtration barrier. Proteoglycans bearing sulfated glycosaminoglycan chains create a dense electronegative meshwork that repels similarly charged macromolecules, reducing their effective filtration coefficient. The endothelial glycocalyx and glomerular basement membrane have historically been considered the primary contributors to this charge selectivity [7,54]. However, recent evidence suggests the podocyte slit diaphragm also possesses electrostatic properties that contribute to charge-based discrimination [55].

Despite clear experimental demonstration of charge effects, the relative physiological importance of charge selectivity remains actively debated. Classic studies using differentially charged dextrans—glucose polymers produced by certain bacteria—or proteins showed that cationic molecules traverse the barrier more readily than anionic ones of equivalent size. However, genetic models that prevented podocyte heparan sulfate synthesis—dramatically reducing barrier negative charge—caused only mild albuminuria despite profound charge alterations [56,57]. These findings suggest that, while charge selectivity exists and can be demonstrated experimentally, size selectivity may dominate under physiological conditions for most proteins.

The clinical implications are nonetheless significant. Proteins with substantial negative charge density, particularly those heavily sialylated or carrying acidic amino acid patches, benefit from enhanced electrostatic repulsion that complements size-based retention. This principle has been exploited therapeutically through glycoengineering strategies that increase sialic acid content (Section 3.4) and it informs the design of long-circulating protein therapeutics. Conversely, highly cationic therapeutic proteins face accelerated renal clearance and may require neutralization or fusion to larger carriers to achieve acceptable pharmacokinetics.

Overall, the evidence supports electrostatic selectivity as a real but secondary component of glomerular permeability. While tracer studies clearly demonstrate preferential filtration of cationic macromolecules, in vivo models show that changes in barrier charge result in only modest proteinuria when size selectivity is preserved [58,59]. These findings suggest that charge primarily fine-tunes filtration by enhancing retention of anionic proteins and accelerating clearance of cationic species, rather than serving as the dominant physiological barrier. This synthesis reconciles experimental observations with in vivo behavior and explains the continued relevance of charge in therapeutic protein design. Understanding the interplay between size and charge selectivity thus remains essential for rational therapeutic design, even as the field continues to refine its mechanistic understanding of their relative contributions.

#### 2.3.2. Glycosylation and Sialylation

Glycosylation is one of the most widespread and functionally important post-translational modifications in nature. It involves the covalent attachment of carbohydrate structures—glycans—to specific protein residues. In humans, it is estimated that at least 50% of the 20,000 protein-coding genes contain glycosylation motifs [60]. These glycans are synthesized and matured within the endoplasmic reticulum and Golgi apparatus through the coordinated activity of glycosyltransferases and glycosidases. Two major forms predominate in plasma proteins: N-linked glycosylation, where glycans attach to asparagine residues within an Asn-X-Ser/Thr motif, and O-linked glycosylation, where sugars are added to serine or threonine residues. In both cases, glycans are highly hydrated, flexible, and capable of substantially increasing a protein’s hydrodynamic volume.

A defining feature of many mature glycans is the presence of terminal sialic acids, a family of nine-carbon sugars that carry a strong negative charge at physiological pH. The process of adding these residues, known as sialylation, is carried out by sialyltransferases in the Golgi. The degree to which a glycoprotein is sialylated affects not only its net charge but also its susceptibility to receptor-mediated clearance: desialylated glycoproteins are rapidly recognized and removed by hepatic asialoglycoprotein receptors, whereas fully sialylated structures avoid recognition and persist in circulation [61,62].

These biochemical properties translate into survival advantages against renal filtration. Glycans increase the protein’s hydrodynamic radius and steric bulk, thereby reducing its filtration across the glomerular barrier. Indeed, empirical studies demonstrate that size is a major determinant of filtration, and large proteins are more restricted [63]. Simultaneously, terminal sialic acids impart a significant negative charge, which enhances electrostatic repulsion from the negatively charged components of the glomerular filtration barrier. The combined effects of steric enlargement and electronegative shielding allow many glycoproteins to resist filtration more effectively than their non-glycosylated counterparts [17,64,65].

Erythropoietin (EPO) illustrates the influence of glycosylation on pharmacokinetics. Native EPO contains three N-glycans and one O-glycan, with each glycan being capable of carrying multiple sialic acids. Darbepoetin alfa introduces two additional N-glycosylation sites, thereby increasing glycan and sialic acid content. These modifications extend in vivo half-life from 8.5 to 25.3 h by amplifying both hydrodynamic size and negative charge, reducing renal filtration and delaying receptor-mediated clearance [23,66,67].

Endogenous biology provides further reinforcement. Many long-lived serum glycoproteins—such as transferrin, α1-acid glycoprotein—rely heavily on multibranched, sialylated glycans to remain in circulation. In experimental models, enzymatic removal of sialic acids leads to rapid clearance, while increased sialylation increases mean residence tTime [68,69].

Thus, glycosylation and sialylation constitute a central natural survival strategy: they enlarge hydrodynamic size, provide electrostatic repulsion at the filtration barrier, and shield proteins from hepatic clearance pathways. These principles form the biological foundation for the engineered glycoengineering approaches discussed in Section 3.4, where the same mechanisms are leveraged to enhance the pharmacokinetics of therapeutic proteins.

## 3. Engineered Half-Life Extension Strategies

Just as endogenous plasma proteins employ diverse structural adaptations to evade rapid renal clearance, therapeutic biologics have been similarly engineered to mimic these survival strategies. Over the past few decades, multiple technological platforms have emerged—each leveraging a distinct biochemical principle to increase hydrodynamic size, engage recycling receptors, or alter surface charge in ways that attenuate filtration across the glomerular barrier. The following section surveys the major modalities used in contemporary biopharmaceutical design, highlighting their mechanistic basis, clinical maturity, and evolving role within the therapeutic landscape (see Table 1).

### 3.1. PEGylation

**Figure 3.**
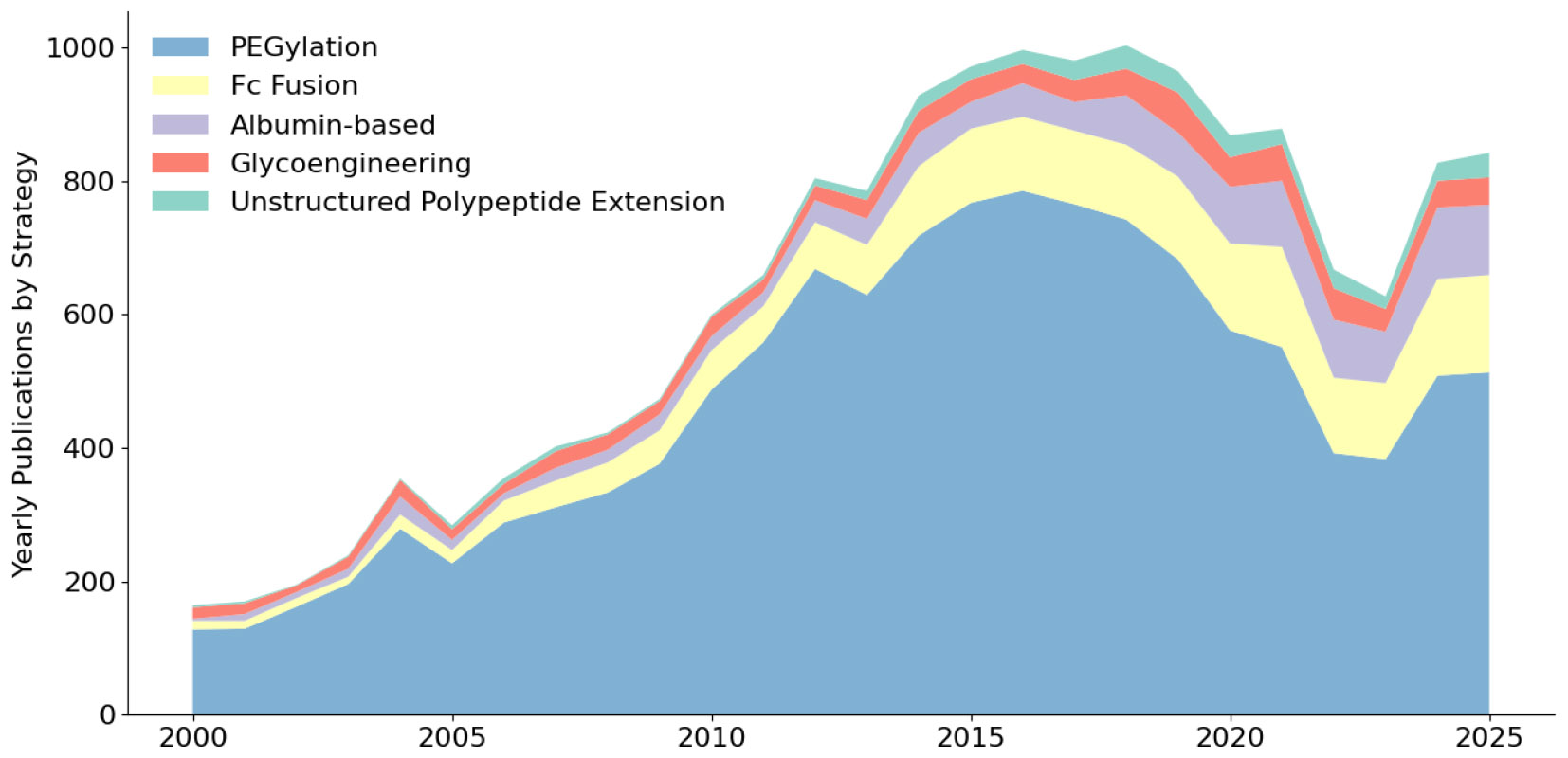
Number of annually published articles including half-life extension strategies from 2000 to 2025 in the PubMed Database, based on search queries detailed in Table A1.

PEGylation is the most thoroughly established method for prolonging the half-life of therapeutics by impeding their renal filtration (see Figure 3). PEGylation enhances pharmacokinetics by covalently attaching polyethylene glycol (PEG) chains to therapeutic proteins, increasing hydrodynamic size and thereby reducing renal filtration [75,76]. The hydrated PEG corona forms a flexible steric barrier that shields proteins from proteolysis, receptor-mediated uptake, and immune recognition [77,78]. Linear PEGs between 5 and 60 kDa remain common, while branched architectures achieve comparable hydrodynamic radii with lower polymer mass, providing additional control over size expansion and solvation behavior [79,80].

These physicochemical properties have led to clinically successful, long-acting biologics. Pegfilgrastim extends G-CSF half-life from ~3.5 h to 15–80 h. Similarly, Peginterferon alfa permits once-weekly dosing, while PEG-asparaginase supports biweekly dosing—a significant reduction from the two to three doses per week required for the un-PEGylated version [81-84].

The same steric features that limit filtration can restrict distribution. Increased hydrodynamic radius slows extravasation and diffusion through dense or poorly vascularized tissues, creating a circulation–penetration trade-off analogous to other macromolecular scaffolds [85-87]. The magnitude of this effect scales with PEG size and architecture, requiring careful selection of polymer mass to balance filtration resistance with target accessibility.

PEGylation can also diminish specific activity. Random conjugation generates heterogeneous species in which the polymer may partially occlude receptor-binding epitopes or perturb essential conformational dynamics, producing measurable reductions in potency [88-91]. Site-specific PEGylation approaches mitigate these impacts by positioning the polymer away from functional interfaces [92].

Immunogenicity additionally influences the performance of PEG-modified therapeutics. Pre-existing anti-PEG antibodies can accelerate clearance or trigger hypersensitivity reactions, with clinical examples including pegloticase and the anti-PEG immunoglobin E–associated responses observed for pegnivacogin [93-97]. As PEG use increases, these antibody-mediated interactions have become important considerations for predicting exposure and clinical variability.

Manufacturing and regulatory constraints also shape PEGylation strategy. Chemical conjugation requires stringent control over PEG stoichiometry and positional selectivity to maintain batch uniformity [18,98]. These requirements, combined with PEG’s non-biodegradable nature, impose analytical and safety considerations that scale with polymer size and dosing frequency. Regulatory classification can introduce further complexity, as PEGylated constructs may be evaluated as combination products requiring additional safety assessments [99]. As a result, PEGylated constructs must be engineered with precise control over chain length, conjugation site, and modification density to achieve predictable pharmacokinetic behavior without introducing excessive heterogeneity or long-term polymer burden.

### 3.2. Fc Fusion Proteins

Fc fusion proteins are created by genetically linking a therapeutic protein, peptide, or receptor domain to the constant (Fc) region of an immunoglobulin G antibody, thereby conferring upon the payload the favorable pharmacokinetic properties inherent to the Fc domain [100-102]. The resulting chimeric molecule simultaneously acquires substantial hydrodynamic size (typically 100–150 kDa in its native dimeric form), pH-dependent binding to the neonatal Fc receptor (FcRn) that enables efficient recycling and protection from lysosomal degradation, and, in many cases, enhanced target binding through enforced dimerization [101,103,104]. These combined attributes transform rapidly cleared biologics into long-circulating therapeutics capable of sustained systemic exposure and reduced dosing frequency.

This approach has yielded several cornerstone medicines that exemplify the clinical translation of FcRn biology. Etanercept, a TNFR2-Fc fusion, sustains twice-weekly dosing for rheumatoid arthritis and related conditions [105]. Abatacept (CTLA-4Ig), employed in rheumatoid arthritis, exhibits terminal half-lives of 13–16 days [106]. Aflibercept (Vascular Endothelial Growth Factor (VEGF) Trap-Fc) extends intravitreal VEGF neutralization to monthly or longer intervals in retinal disease [107]. Romiplostim, a thrombopoietin mimetic peptide fused to Fc, supports weekly or less frequent administration in chronic immune thrombocytopenia [108]. Across these diverse payloads—receptor ectodomains, cytokines, and peptides—the common Fc module provides predictable pharmacokinetic extension while leveraging decades of manufacturing experience with monoclonal antibodies [19,104,109].

The platform’s versatility is further enhanced by protein engineering. Effector functions (antibody-dependent cellular toxicity, complement-dependent cytotoxicity, complement activation) can be abrogated through targeted mutations (e.g., LALA or N297 substitutions) to create “silenced” Fc backbones when only pharmacokinetic extension is desired [110]. Conversely, affinity for FcRn can be deliberately augmented at acidic pH (e.g., YTE or LS mutations), yielding half-lives that approach or exceed those of native IgG and demonstrably improving in vivo potency [111,112]. Monomeric Fc variants have also been explored, offering intermediate molecular weights (~75 kDa) that may better balance circulation time with tissue penetration in certain contexts [100].

Nevertheless, the very attributes conferring longevity introduce trade-offs. The large hydrodynamic volume of dimeric Fc fusions can impede diffusion into poorly vascularized or densely structured tissues, a limitation particularly relevant for solid tumor targeting or delivery to the central nervous system [113]. Unwanted immunological effector activity, when not adequately silenced, may provoke inflammatory side-effects or accelerated clearance in some patients. Additionally, expression yields of Fc -fusion proteins are frequently lower than those of monoclonal antibodies, reflecting the added biosynthetic burden of the fused domain [114]. These considerations, while not prohibitive, underscore the continuing need for rational design that tailors Fc architecture—dimeric versus monomeric, wild-type versus engineered—to the specific therapeutic challenge at hand.

Thus, Fc fusion remains one of the most mature and successful translations of nature’s pharmacokinetic blueprint, delivering proven clinical benefit while continually evolving through structural insight and mutational refinement [19,104].

### 3.3. Albumin-Based Strategies

Serum albumin (66.5 kDa, ~35–50 g/L) achieves its ~19–21-day half-life through a combination of its large hydrodynamic size and highly efficient pH-dependent FcRn-mediated recycling, the same salvage pathway exploited by IgG [115-117]. Its abundance, lack of immunogenicity, and extended systemic duration have made albumin the most widely used endogenous carrier for engineered half-life extension [118]. Three distinct strategies have reached clinical practice.

First, direct albumin fusion: In this strategy, the therapeutic protein is genetically fused to albumin. This fusion increases molecular weight to surpass the glomerular filtration threshold and directly confers FcRn recycling. Albiglutide, a glucagon-like-peptide-1(GLP-1)–albumin fusion, was the first approved example and enabled once-weekly dosing in type 2 diabetes [119].

Second, fatty-acid acylation: Albumin possesses multiple hydrophobic pockets that naturally bind fatty acids with high affinity. By chemically attaching a saturated fatty acid chain—typically C14–C20—to a peptide or protein therapeutic, the modified drug associates reversibly with endogenous albumin in plasma. Two therapeutics that exemplify this strategy are Myristoylated insulin detemir and di-fatty-acid-modified insulin degludec, which bind to circulating albumin with greater than 98% occupancy, extending the duration of action up to 42 h and forming the basis of modern ultra-long-acting insulins [120,121].

Third, Albumin-binding domains (ABDs) are small (~5–15 kDa) high-affinity polypeptides derived from bacterial proteins or de novo design. Therapeutics are genetically fused to these binding domains, which in turn bind to Albumin [113,122]. The resulting construct remains compact (typically 30–80 kDa) yet inherits near-complete FcRn protection upon reversible binding to endogenous albumin. Ozoralizumab, a trivalent anti-TNFα nanobody carrying an ABD (~40–50 kDa total), was approved in Japan (2022) for rheumatoid arthritis with once- or twice-monthly subcutaneous dosing, demonstrating superior tissue penetration compared with 150 kDa Fc-fused TNF inhibitors while achieving comparable pharmacokinetic extension [123].

Relative to dimeric Fc fusions, ABD constructs occupy an attractive intermediate size range: when albumin-bound they are effectively >200 kDa in hydrodynamic volume, but the unbound fraction retains the diffusion properties of a small protein, offering a favorable balance between circulatory persistence and extravascular access [124,125].

Limitations include occasional steric interference with target engagement, non-linear pharmacokinetics due to reversible binding, and marked species differences in albumin sequence that often weaken ABD affinity in preclinical models and complicate translational PK [13,126]. Despite these challenges, albumin-based technologies have yielded at least nine approved therapeutics and remain one of the most versatile, clinically validated platforms for extending the half-life of peptides, nanobodies, and other small biologics (see Table A2) [127].

### 3.4. Glycoengineering and Polysialic Acid

Glycoengineering encompasses the rational modification of protein glycosylation patterns to enhance pharmacokinetics while preserving biological function [109,128,129]. This approach applies natural principles—particularly sialylation-dependent half-life extension—systematically to therapeutics through genetic, enzymatic, or chemical methods. By manipulating glycosylation site occupancy, glycan composition, branching, and sialylation degree, glycoengineering offers precise pharmacokinetic tuning, though structural complexity creates unique technical and regulatory challenges.

The most straightforward approach introduces additional N-glycosylation sites through site-directed mutagenesis of consensus sequences (Asn-X-Ser/Thr). Darbepoetin alfa exemplifies this success, though site selection critically depends on surface exposure and distance from functional interfaces to avoid activity loss. Expression system selection provides another powerful lever: Chinese hamster ovary (CHO) cells, human cell lines, yeast, and insect cells produce characteristically different glycosylation patterns reflecting their endogenous glycosyltransferase repertoires [129]. CHO cells dominate therapeutic production due to their safety profile and capacity for complex, sialylated glycan synthesis, though recent glycoengineering enables “humanization” of non-mammalian systems through transgenic glycosyltransferase expression.

Controlling glycan branching and sialylation represents more sophisticated engineering. N-glycan antennae generated by specific glycosyltransferases (particularly GnT-V) can each be terminated with sialic acids. Tetra-antennary glycans with maximal sialylation carry more negative charge and occupy larger hydrodynamic volumes than bi-antennary structures. This has been exploited through overexpressing branching enzymes, modulating sialyltransferase levels, or optimizing culture conditions. Hypersialylated glycoproteins demonstrate extended half-lives approaching or exceeding PEGylation while maintaining biological degradability.

Polysialic acid (PSA) conjugation—inspired by neural cell adhesion molecules carrying long α2,8-linked sialic acid chains (20–200 residues)—offers biomimetic half-life extension [130,131]. These linear homopolymers generate exceptional negative charge density and hydrodynamic volume relative to mass, with theoretical advantages over PEG: superior filtration resistance per unit mass, strong electrostatic repulsion, and biodegradability through endogenous neuraminidases. Preclinical studies demonstrate PSA-modified proteins achieve pharmacokinetic improvements comparable to PEGylation.

However, PSA faces significant translational barriers limiting clinical adoption [132]. Enzymatic or chemical PSA synthesis with controlled length distribution remains technically challenging and expensive versus mature PEG infrastructure. Chemical conjugation must address inherent heterogeneity while ensuring site-specific attachment. High manufacturing costs, complex quality control, and limited regulatory precedent have relegated PSA to exploratory status despite theoretical advantages.

The heterogeneity inherent to glycosylation—natural or engineered—presents persistent analytical and regulatory challenges [133,134]. Microheterogeneity in site occupancy, branching, and terminal modifications complicate batch consistency, requiring sophisticated analytical methods and detailed regulatory characterization.

### 3.5. Unstructured Polypeptide Extensions

Unstructured polypeptide extensions encompass the genetic fusion of hydrophilic, intrinsically disordered amino acid sequences to therapeutic proteins, thereby enhancing pharmacokinetics through size expansion while minimizing impacts on biological function [22,135]. This strategy draws from natural principles of hydrodynamic augmentation but achieves effects via recombinant polypeptide chains composed of non-immunogenic residues such as proline, alanine, serine, glycine, threonine, and glutamate. By appending sequences typically ranging from 100 to 1000 residues, unstructured extensions offer tunable half-life prolongation, with applications in genetic, enzymatic, or chemical fusion methods. XTEN, PAS (proline-alanine-serine), and HAP (homo-alanine polymer) represent the primary platforms, each providing precise control over molecular dimensions, though sequence composition and length introduce distinct biophysical and regulatory considerations [135,136].

The foundational approach involves site-directed genetic fusion, wherein the polypeptide extension is appended to the N- or C-terminus—or, less commonly, inserted internally—via standard recombinant DNA techniques. Expression systems play a critical role: *E. coli* and other microbial hosts enable high-yield production due to the sequences’ lack of complex post-translational requirements, while mammalian systems such as CHO cells may be employed for proteins necessitating glycosylation or disulfide bonding [103]. Unlike globular proteins, the designed extensions lack stable secondary or tertiary structure, minimizing hydrophobic interactions and aggregation risks [137,138]. Additionally, their hydrophilic nature enhances solubility and serum stability, reducing proteolytic degradation and immunogenicity compared to synthetic polymers like PEG [138].

XTEN technology exemplifies sophisticated engineering within this class. Comprising repeats of alanine, glutamate, glycine, proline, serine, and threonine, XTEN sequences form highly soluble, unstructured polymers that confer exceptional filtration resistance. Preclinical studies demonstrate half-life extensions of 10- to 100-fold for fused peptides and proteins, as seen with exenatide-XTEN (half-life increased from ~2 h to >100 h in rodents) and GLP-2-XTEN analogs for gastrointestinal disorders [139,140]. Clinically advanced XTEN fusion therapeutics include VRS-317 (somavaratan), an XTEN-extended human growth hormone that demonstrated markedly prolonged serum half-life and once-weekly to twice-monthly dosing potential in early-phase trials [141,142]. In 2023, efanesoctocog alfa, a factor VIII replacement that incorporates Fc, VWF(Von Willebrand Factor)-D’D3, and XTEN polypeptides, was approved by the FDA for once-weekly prophylaxis in hemophilia A. Clinical pharmacokinetics show a terminal half-life in the range of ~40 to 48 h—approximately three- to four-fold longer than standard or earlier extended half-life FVIII products [143,144].

PASylation offers a complementary platform, utilizing randomized proline-alanine-serine repeats to generate biodegradable extensions with tunable lengths. This approach has prolonged circulation for diverse biologics, including cytokines and antibody fragments [138]. For instance, PASylated interferon alpha demonstrated a ~29-fold half-life increase in mice, while PAS-fused leptin extended terminal half-life from ~0.5 h to ~20 h, supporting potential obesity treatments [138,145]. Growth hormone PASylation similarly yielded 90-fold extensions [138].

HAP sequences, composed of glycine-rich homo-amino-acid polymers—specifically, repetitive (Gly_4_Ser)_n_ motifs—represent a simple yet effective variant focused on poly-alanine chains of 100–200 residues. Though less diverse than XTEN or PAS, HAPylation increases hydrodynamic volume via long, flexible, solvated polypeptide chains, thereby reducing renal clearance. Proof-of-concept studies with HAP-fused Fab fragments revealed half-life extensions from ~0.14 h to ~0.6 h in rodents, demonstrating size-dependent renal retention [22]. While HAP has seen limited clinical advancement compared to its counterparts, its application to enzymes and peptides underscores the potential for cost-effective microbial production and minimal immunogenicity.

Despite these successes, challenges persist in balancing extension length with therapeutic performance. Excessive size can impair tissue penetration, particularly in dense matrices like tumors, mirroring limitations of larger fusions [146]. Fusion site selection is critical to avoid occluding active domains or altering conformational dynamics, which may reduce potency; for example, internal insertions risk destabilization, necessitating empirical optimization [147]. Immunogenicity, while generally low, remains uncertain for long-term use, with potential T-cell epitopes in repetitive sequences requiring deimmunization strategies [148]. Regulatory hurdles arise from product heterogeneity if extensions vary in length, demanding advanced analytics for consistency [149]. As with glycoengineering, these extensions must address species-specific pharmacokinetics during preclinical translation, given differences in renal clearance across models [150].

### 3.6. Immunogenic Risk Profiles

A common challenge in extending the circulating half-life of therapeutics is the risk of immunogenic responses, which generally increases with the degree of structural deviation from endogenous molecules. Although PEGylation was initially considered non-immunogenic, accumulating evidence demonstrates the formation of anti-PEG antibodies that can accelerate clearance and reduce therapeutic efficacy, particularly upon repeated dosing, where antibody titers may increase over time [151,152]. Consequently, patient screening and immunogenicity monitoring may be important considerations for PEGylated biologics, especially in chronic treatment settings. In contrast, albumin-based strategies and Fc fusion proteins typically exhibit low immunogenic risk, as both human serum albumin and the IgG Fc region are endogenous. Anti-drug antibody incidence is generally low, and antibodies are most often non-neutralizing with minimal impact on pharmacokinetics or efficacy, although albumin-binding domains derived from bacterial proteins represent a notable exception due to their potential to elicit immune responses [27,153]. The immunogenicity of glycoengineered biologics is highly dependent on glycan composition, with human-like glycans being well tolerated, whereas non-human sugars such as α-galactose or N-glycolylneuraminic acid can provoke strong anti-drug antibody or hypersensitivity reactions [154]. Finally, unstructured polypeptide extensions are intentionally designed to minimize immunogenicity by avoiding sequence motifs associated with immune recognition and have not been observed to induce significant anti-drug antibody formation in preclinical and early clinical studies [139].

## 4. Emerging Strategies

### 4.1. Homodimerization

Proteins in nature frequently assemble into complexes in which two or more identical subunits associate to form homodimers or higher-order oligomers. This quaternary organization confers numerous advantages for protein stability and function, including an increased hydrodynamic radius [155] which allows proteins that would otherwise be filtered by the glomerulus to remain in circulation. The protein engineering community has leveraged this natural mechanism in various designs [156-158]. By engineering interfaces that promote attraction between identical subunits, researchers can induce proteins to self-assemble into larger complexes. Several strategies have been employed to design these protein–protein interfaces, including β-strand extension, coiled-coil architectures, and covalent stabilization through engineered disulfide bonds or chemical crosslinks [159,160].

Synthetic peptide homodimers targeting the VEGF demonstrated that tuning the linker length between homodimers and adjusting the distance between them improves both avidity and anti-angiogenic potency, while simultaneously increasing hydrodynamic size [161]. Another application is dimeric peptides that target the G-protein-coupled receptor CXCR4. Some dimeric peptides targeting CXCR4 have shown increased in vivo persistence relative to monomeric forms, likely due to increased steric bulk and reduced filtration rates [157].

Beyond benefitting from an increased hydrodynamic radius, homodimerized proteins can have improved conformational stability [162,163]. Another important feature of homodimerization is that it bypasses the need for adding synthetic polymers onto the protein, thereby reducing the risk for immunogenicity [164]. Successful application of this method requires careful engineering. Over-stabilization may reduce receptor accessibility, while insufficient stability can lead to dissociation and decreased pharmacokinetics.

Overall, homodimerization represents a promising approach for half-life extension. By leveraging an evolutionary mechanism to increase the hydrodynamic size of a protein complex, homodimers provide a fully protein-based alternative to methods that require synthetic polymers or complex post-translational modifications.

### 4.2. De Novo Protein Design

While the half-life extension strategies from Section 3 accomplish their purpose by increasing the effective hydrodynamic radius, these approaches typically rely on post-translational modifications or fusions to existing domains rather than intrinsic structural design. Such approaches often introduce heterogeneity, complicate manufacturing, and pose immunogenicity risks—challenges notably seen with anti-PEG antibodies or chemically conjugated products [152,165,166]. Recent research has shown a strong emphasis on de novo protein design, which focuses on producing a single biological entity instead of relying on extrinsic modifications. This structural simplicity streamlines the manufacturing process and allows for atomic-level precision in avoiding steric occlusion of the active site, thereby reducing the activity-stability trade-offs often associated with carrier-based strategies [26]. The de novo design process can integrate a variety of natural half-life extension mechanisms into the protein scaffold.

Despite these advantages, the clinical translation of de novo designed therapeutics remains in its nascent stages. A primary challenge lies in immunogenicity; while computational methods can screen for known epitopes, novel sequences possess the risk of eliciting anti-drug antibodies upon repeated dosing [167]. Furthermore, it can be difficult to ensure solubility and prevent aggregation at high clinical concentrations, even after computational optimization [168]. A common critique of de novo designed scaffolds is the stability-activity tradeoff. Backbones designed de novo tend to exhibit impressive stability. However, this structural rigidity can impede the dynamics needed for efficient catalysis [160]. While challenges such as immunogenicity, solubility, and the stability-activity tradeoff remain, de novo design offers versatile strategies that will be explored to highlight its substantial potential for clinical application.

#### 4.2.1. Supercharging

Surface charge has long been considered a contributor to renal filtration due to the negatively charged heparan sulfate proteoglycans coating the glomerular filtration barrier, although the extent of charge selectivity for proteins remains actively debated in vivo [7]. Nevertheless, engineering proteins with increased net negative charge can influence clearance in certain contexts, as demonstrated in protein-surface engineering studies [169-171]. De novo design enables this charge modification by allowing negative residues to be incorporated more frequently in solvent-exposed positions while preserving core stability [172]. While supercharging alone is unlikely to prevent filtration without sufficient size, it is a tunable design parameter that can be integrated with other strategies to modulate pharmacokinetics more predictably.

#### 4.2.2. Novel Binding Domains

Utilizing endogenous recycling pathways, such as albumin or FcRn, is an established method for extending half-life [173]. Traditional approaches rely on natural binding domains or screening large amounts of proteins/peptides to identify high-affinity motifs. De novo generative models offer a route to design compact binding domains directly against albumin or Fc epitopes, using conditional diffusion models for backbone generation and inverse-folding networks for sequence optimization [20,174,175]. These methods reduce dependence on broad library screening while enabling atomic-level control over binding geometry. Although experimental validation of affinity and function remains essential, de novo binders are an exciting new area of study and possess the potential to save substantial resources.

#### 4.2.3. Modified Immunogenicity

A significant risk posed by many biologics is immunogenicity [176]. Proteins can trigger anti-drug antibody responses, which neutralize the drug or accelerate its clearance through immune complex formation. Recent models identify and reduce epitopes that are likely to be detected by the body’s immune system [177]. These computational filters do not eliminate immunogenicity but serve as an early-stage safeguard for selecting sequences with lower predicted T-cell activation potential. Incorporating these constraints during backbone and sequence generation allows protein design to balance characteristics like thermodynamic stability and catalytic activity with reduced immunological risk, improving the likelihood of favorable pharmacokinetics during preclinical development.

#### 4.2.4. Hydrodynamic Radius

A significant methodological gap currently exists in the field: the inability to directly and reliably enforce a specific hydrodynamic radius, *R_h_*, or molecular weight as a primary goal during the de novo generative design process. To date, no published methodology has integrated hydrodynamic size constraints directly into generative models to explicitly engineer proteins that avoid renal filtration, representing an unexplored area in protein engineering.

The theoretical approach to achieve this is well established, as state-of-the-art diffusion-based protein design models already have the necessary capabilities for constraint-based generation. These models enable sophisticated modeling, conditional generation, and guided sampling based on complex structural features, including specific sequence motifs, binding geometries, and defined oligomeric states [178-188]. In principle, these same conditioning or guidance frameworks can be extended to incorporate *R_h_* or the radius of gyration *R_g_* as explicit design constraints. By setting a strict lower limit for these physical dimensions—aiming for an *R_h_* greater than the approximate 3 nm threshold needed for retention—generative models could be directed to sample only protein scaffolds that naturally surpass the glomerular cutoff without requiring external chemical modifications [189].

## 5. Conclusions

Therapeutic biologics face the same physiological constraints that have shaped endogenous plasma proteins, and the kidney remains a central determinant of circulatory fate. As this review outlines, nature possesses a repertoire of structural and biochemical strategies—from increased hydrodynamic size to receptor-mediated salvage—to prolong systemic circulation.

Engineered half-life extension technologies build off these principles, translating evolutionary solutions into platforms capable of improving dosing convenience, therapeutic exposure, and overall clinical feasibility. Although established methods such as PEGylation and Fc-fusion have been dominant for decades, newer approaches—such as albumin fusion, glycoengineering, and emerging polypeptide-based systems—are diversifying the landscape of protein therapeutics and offer alternatives with improved biodegradability, tunability, and potentially reduced immunogenicity.

While newer methods have demonstrated immense potential to create more effective therapeutics, they face higher barriers to entry due to unproven safety profiles and a lack of long-tern clinical data. Pharmaceutical developers are often hesitant to invest substantial resources into these emerging technologies when established platforms offer a more predictable path to regulatory approval. Consequently, commercial entities may favor validated modalities to mitigate the risks associated with less-proven ones. Other challenges on the scientific front remain, such as balancing circulation time with tissue penetration, managing immunogenicity risks, preserving biological activity, and controlling manufacturing complexity. Continued integration of physiological insight, molecular engineering, machine learning, and clinical validation will be essential for refining these platforms and developing next-generation therapeutics that more effectively navigate the constraints of renal clearance.

## Figures and Tables

**Table 1.** Summary of major half-life extension strategies used in clinical and preclinical biologics.

Strategy	Molecular Weight Increase	Best-Suited Targets	# of FDA-Approved Therapeutics	Year of First FDA Approval
PEGylation	+5–40 kDa	Proteins/peptides sensitive to clearance, enzymes in need of longer dosing intervals	26	1990 [70]
Fc Fusion Proteins	+50–70 kDa	Cytokines, receptors, peptides that tolerate C- or N- terminal fusion	12	1998 [71]
Albumin-Based Strategies	+65 kDa	Small peptides with short half-lives, coagulation factors, hormones	9	2005 [72]
Glycoengineering	+2–10 kDa	Antibodies, Fc-containing molecules, proteins that rely on FcRn	1	2001 [73]
XTEN	+12–72 kDa	Peptides, enzymes, and cytokines that tolerate long flexible tails	1	2023 [74]

## Data Availability

No new data were created or analyzed in this study. Data sharing is not applicable to this article.

## Appendix A

### Appendix A.1

**Table A1.** Boolean search strings used to determine the historical relevance of PEGylation, Fc-fusion, albumin-binding, glycoengineering, and unstructured polypeptide extensions. Each query was constructed in two parts: (1) a comprehensive set of synonyms defining the specific engineering strategy, and (2) a fixed set of search terms regarding half-life and clearance (the “AND” section), which was maintained across all queries to ensure comparability.

Strategy	Boolean Search String
PEGylation	(PEGylation OR PEGylated OR PEGylate OR “PEG-modified” OR “PEG-conjugated” OR “polyethylene glycol” OR “poly(ethylene glycol)”) AND (half-life OR “half life” OR “extended half-life” OR “half-life extension” OR “circulation time” OR “increased circulation” OR clearance OR “renal clearance” OR “renal filtration” OR “pharmacokinetics” OR PK OR “biodistribution”)
Fc-fusion	(“Fc fusion” OR “Fc-fusion” OR “Fc-fused” OR “Fc-conjugated” OR “IgG Fc” OR “Fc domain” OR “immunoglobulin Fc” OR FcRn) AND (half-life OR “half life” OR “extended half-life” OR “half-life extension” OR “circulation time” OR “increased circulation” OR clearance OR “renal clearance” OR “renal filtration” OR “pharmacokinetics” OR PK OR “biodistribution”)
Albumin-binding	(“albumin fusion” OR “albumin-fused” OR “albumin-binding” OR “albumin binding domain” OR ABD OR “albumin-binding peptide” OR “HSA fusion” OR “human serum albumin fusion” OR “albumin conjugation” OR “albumin hitchhiking”) AND (half-life OR “half life” OR “extended half-life” OR “half-life extension” OR “circulation time” OR “increased circulation” OR clearance OR “renal clearance” OR “renal filtration” OR “pharmacokinetics” OR PK OR “biodistribution”)
Glycoengineering	(glycoengineering OR glycan engineering” OR “glycan remodeling” OR sialylation OR “terminal sialic acid” OR polysialic OR polysialylation OR polysialylated) AND (half-life OR “half life” OR “extended half-life” OR “half-life extension” OR “circulation time” OR “increased circulation” OR clearance OR “renal clearance” OR “renal filtration” OR “pharmacokinetics” OR PK OR “biodistribution”)
Unstructured Polypeptide Extensions	(PASylation OR “PAS polypeptide” OR “PAS sequence” OR PAS-tag OR XTEN OR XTENylation OR “unstructured polypeptide” OR “disordered polypeptide” OR “intrinsically disordered extension” OR IDP OR “elastin-like polypeptide” OR ELP) AND (half-life OR “half life” OR “extended half-life” OR “half-life extension” OR “circulation time” OR “increased circulation” OR clearance OR “renal clearance” OR “renal filtration” OR “pharmacokinetics” OR PK OR “biodistribution”)

## Appendix B

### Appendix B.1

**Table A2.** Table of FDA-approved therapeutics which utilize one of the following half-life extension strategies: PEGylation, Fc-fusion, albumin-binding, glycoengineering, or unstructured polypeptide extensions. These include therapeutics that have been discontinued.

PEGylation	Fc-Fusion	Albumin-Binding	Glycoengineering	Unstructured Polypeptide Extensions
Palopegteriparatide [190]	Sotatercept [191]	Tirzepatide (Mounjaro) [192]	Darbepoetin alfa [193]	Efanesoctocog alfa [144]
Pegulicianine [194]	Dulaglutide [195]	Tirzepatide (Zepbound) [196]		
Pegunigalsidase alfa [197]	Efmoroctocog alfa [198]	Semaglutide [199]		
Pegfilgrastim-pbbk [200]	Eftrenonacog alfa [201]	Albutrepenonacog alfa [202]		
Pegfilgrastim-fpgk [203]	Ziv-aflibercept [204]	Insulin degludec [121]		
Lonapegsomatropin [205]	Aflibercept [206]	Liraglutide (Saxenda) [207]		
Ropeginterferon alfa-2b-njft [208]	Belatacept [209]	Liraglutide (Victoza) [210]		
Pegfilgrastim-apgf [211]	Romiplostim [212]	Albiglutide [213]		
Turoctocog alfa pegol [214]	Rilonacept [215]	Insulin detemir [216]		
Pegfilgrastim-bmez [217]	Abatacept [218]			
Damoctocog alfa pegol [219]	Alefacept [220]			
Calaspargase pegol-mknl [221]	Etanercept [222]			
Pegfilgrastim-jmdb [223]				
Pegfilgrastim-cbqv [224]				
Nonacog beta pegol [225]				
Rurioctocog alfa pegol [225]				
Peginterferon beta-1a [226]				
Peginterferon alfa-2b [227]				
Certolizumab pegol [228]				
Methoxy polyethylene glycol-epoetin beta [229]				
Pegvisomant [230]				
Pegfilgrastim [200]				
Peginterferon alfa-2a [231]				
Pegaspargase [232]				
Pegademase bovine [233]				

## Abbreviations

The following abbreviations are used in this manuscript:

GFB: Glomerular filtration barrier
IGF: Insulin-like growth factor
IGFBP: Insulin-like growth factor binding protein
ALS: Acid-labile subunit
IgG: Immunoglobulin G
FcRn: Neonatal Fc receptor
EPO: Erythropoietin
PEG: Polyethylene glycol
G-CSF: Granulocyte colony-stimulating factor
TNFR2: Tumor Necrosis Factor Receptor 2
CTLA-4: Cytotoxic T-lymphocyte-associated protein 4
VEGF: Vascular endothelial growth factor
GLP: Glucagon-like peptide
ABD: Albumin-binding domain
CHO: Chinese hamster ovary
PSA: Polysialic acid
PAS: Proline-alanine-serine
HAP: Homo-alanine polymer
VWF: Von Willebrand factor
